# NIK Is a Mediator of Inflammation and Intimal Hyperplasia in Endothelial Denudation-Induced Vascular Injury

**DOI:** 10.3390/ijms252111473

**Published:** 2024-10-25

**Authors:** Ciro Baeza, Marta Ribagorda, Carla Maya-Lopez, Manuel Fresno, Tania Sanchez-Diaz, Aranzazu Pintor-Chocano, Ana B. Sanz, Susana Carrasco, Alberto Ortiz, Maria Dolores Sanchez-Niño

**Affiliations:** 1 Department of Vascular Surgery, IIS-Fundacion Jimenez Diaz UAM, 28040 Madrid, Spain; 2 RICORS2040, 28040 Madrid, Spain; 3 Department of Nephrology and Hypertension, IIS-Fundacion Jimenez Diaz UAM, 28040 Madrid, Spainpintorarancha@hotmail.com (A.P.-C.); 4 Centro de Biología Molecular Severo Ochoa, Consejo Superior de Investigaciones Científicas de la Universidad Autonoma de Madrid, 28049 Madrid, Spain; mfresno@cbm.csic.es; 5 Departamento de Farmacología, Facultad de Medicina, Universidad Autónoma de Madrid, 28049 Madrid, Spain; 6 Departamento de Medicina, Facultad de Medicina, Universidad Autónoma de Madrid, 28049 Madrid, Spain

**Keywords:** NFκB, NFκB-inducing kinase (NIK, MAP3K14), neointimal hyperplasia, endothelial cell injury, atherosclerosis, cardiovascular

## Abstract

Neointimal hyperplasia is the main cause of vascular graft failure in the medium term. NFκB is a key mediator of inflammation that is activated during neointimal hyperplasia following endothelial injury. However, the molecular mechanisms involved in NFκB activation are poorly understood. NFκB may be activated through canonical (transient) and non-canonical (persistent) pathways. NFκB-inducing kinase (NIK, MAP3K14) is the upstream kinase of the non-canonical pathway. We have now explored the impact of NIK deficiency on neointimal hyperplasia following guidewire-induced endothelial cell injury and on local inflammation by comparing NIK activity–deficient alymphoplasia mice (NIK^aly/aly^) with control wild-type (NIK^+/+^) mice. Guidewire-induced endothelial cell injury caused neointimal hyperplasia and luminal stenosis and upregulated the local expression of NIK and the NFκB target chemokines monocyte chemoattractant protein-1 (MCP-1/CCL2) and chemokine ligand 5 (RANTES/CCL5). Immunohistochemistry disclosed the infiltration of the media and intima by F4/80 positive macrophages. The intima/media ratio and percentage of stenosis were milder in the NIK^aly/aly^ than in the NIK^+/+^ mice. Additionally, the gene expression for MCP-1 and RANTES was lower and F4/80+ cell infiltration was milder in the NIK^aly/aly^ than in the NIK^+/+^ mice. Finally, circulating MCP-1 levels were lower in the NIK^aly/aly^ than in the NIK^+/+^ mice, reflecting milder systemic inflammation. In conclusion, NIK is a driver of vascular wall inflammation and stenosis following guidewire-induced endothelial cell injury. NIK targeting may be a novel therapeutic approach to limit arterial stenosis following endothelial cell injury.

## 1. Introduction

Cardiovascular disease is the most common cause of death worldwide and is expected to remain a top cause of death by 2040 [1]. Intimal hyperplasia is the pathological process that underlies arterial restenosis after endothelial injury and results from the migration of vascular smooth muscle cells (VSMCs) [2,3]. It is the main cause of vascular graft failure in the medium term [4,5,6]. A correct understanding of its molecular mechanisms and modulators may help design novel therapeutic approaches that improve the outcomes of endovascular procedures.

Both local and systemic inflammations promote endothelial dysfunction and vascular injury [7,8]. The nuclear factor kappa-light-chain-enhancer of activated B cells (NFκB) is a transcription factor that promotes inflammation and has been involved in neointimal hyperplasia following endothelial injury [9,10]. The activation of NFκB results in the nuclear translocation and modulation of target gene expression and can proceed either through classical/canonical or alternative/non-canonical and hybrid pathways [10,11]. Activating stimuli converge on the phosphorylation and engagement of the inhibitor of the κB kinase (IKK) signalosome. The inhibitors of κB (IκB) proteins regulate the nuclear translocation and DNA binding of NFκB. IKKs phosphorylate IκBs, marking them for ubiquitination and degradation or processing by proteasomes, thus releasing IκB-bound NFκB. In addition, IKKs regulate transcriptional responses by phosphorylating nuclear NFκB proteins and NFκB-associated proteins. Classical NFκB activation is usually a rapid and transient response to a wide range of stimuli whose main effector is RelA/p50. The alternative NFκB pathway is a more delayed and prolonged response to a smaller range of stimuli resulting in the DNA binding of RelB/p52 complexes. Despite much initial interest in targeting the canonical NFκB pathway, so far, no drug targeting the canonical pathway of NFκB activation has reached the clinic. This may be related to the pleiotropic actions of NFκB that also activate cell survival pathways. Non-canonical NFκB may be a therapeutic target of interest, given its contribution to the chronic maintenance of inflammatory responses [11]. NFκB-inducing kinase (NIK, MAP3K14) is the upstream kinase of the non-canonical pathway. Indeed, mice deficient in NIK activity (e.g., NIK^aly/aly^ mice) are protected from diverse forms of tissue injury [12,13] and small molecule NIK inhibitors are under preclinical development and have so far been shown to protect from tissue injury in murine lupus [14]. Of interest, premature cardiovascular disease is currently the main cause of death in patients with lupus [15]. While many cytokines activate the NFκB canonical pathway, only a limited number of them activate the non-canonical pathway [16]. Of the cytokines known to activate the non-canonical NFκB pathway, TWEAK is known to promote vascular injury [17,18]. The activation of the TWEAK/TWEAK receptor (TWEAKR, also known as Fn14) system depends mainly on the upregulation of Fn14, i.e., upon tissue injury, there is a large increase in Fn14 expression that sensitizes cells to TWEAK concentrations that may remain stable or even decrease [19]. We have now explored the expression and function of NIK in neointimal hyperplasia secondary to endothelial cell injury in mice and report that NIK deficiency decreases local inflammation and neointimal hyperplasia, identifying NIK as a potential therapeutic target in neointimal hyperplasia.

## 2. Results

### 2.1. Increased Expression of Fn14 and NIK Following Endothelial Cell Injury

**Figure 1:**
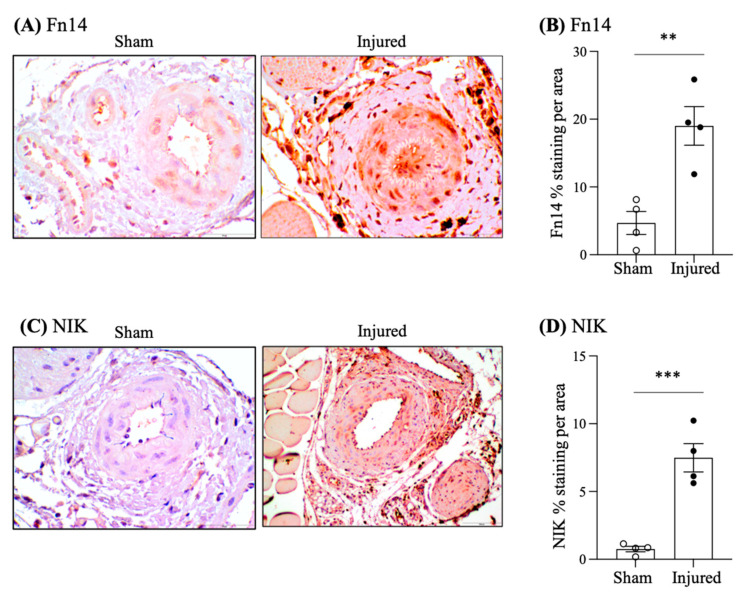
Increased Fn14 and NIK expression in femoral arteries two weeks following endothelial cell injury induced by a guidewire in mice. Guidewire insertion-induced endothelial cell injury in one femoral artery (labeled as “injured” in the figure), while a sham procedure was performed in the contralateral artery of the same mice (labeled as “sham” in the figure). The arteries were studied 14 days later and immunohistochemistry was performed. (**A**,**B**) Increased Fn14 immunostaining following the endothelial cell injury. Representative image (**A**) and quantification (**B**). (**C**,**D**) Increased NIK immunostaining following the endothelial cell injury. Representative image (**C**) and quantification (**D**). ** *p* < 0.005; *** *p* < 0.0005. Original magnification 20x. Mean ± SEM of 4 mice per group.

Endothelial denudation through guidewire-induced injury is an established model of intimal hyperplasia and vascular stenosis. Two weeks after endothelial denudation in mice, an increased expression of Fn14 was observed in cells within the hyperplasia area and in the vascular wall of injured femoral arteries, as compared with the contralateral artery exposed to a sham procedure (Figure 1A,B; sham 4.69 ± 1.69 and injured 19.01 ± 2.85% expression).

An increased immunostaining for the intracellular kinase NIK, an intracellular target of Fn14 that links Fn14 activation to activation of the proinflammatory NFκB transcription factor, was also observed (Figure 1C,D; sham 0.75 ± 0.20 and injured 7.49 ± 1.04% expression).

### 2.2. NIK Deficiency Protected from Arterial Stenosis

**Figure 2:**
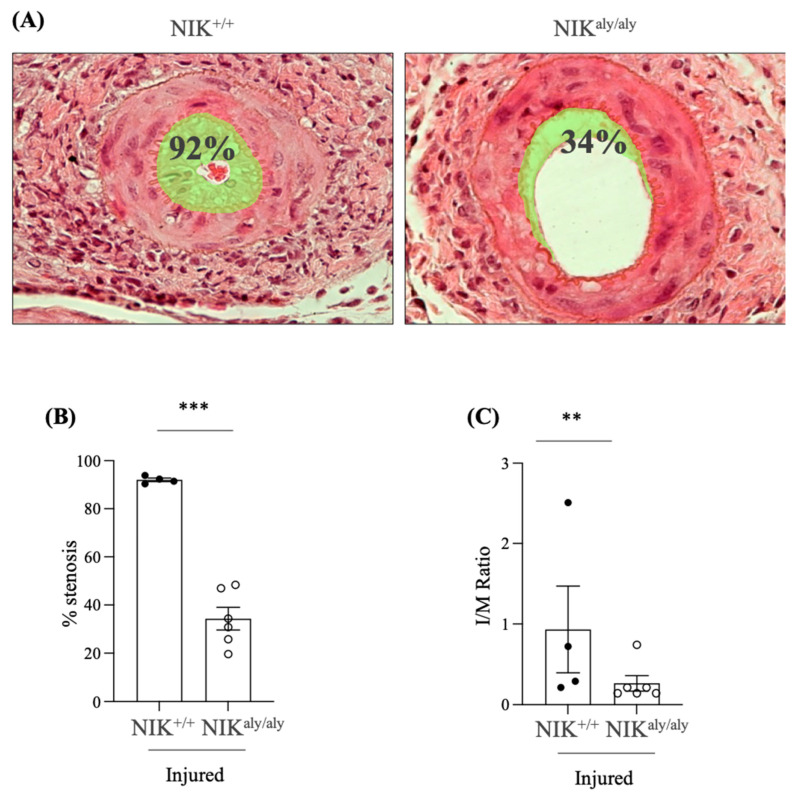
NIK deficiency in NIK^aly/aly^ mice improved the histology of injured femoral arteries two weeks following endothelial cell injury induced by a guidewire. (**A**) HE staining of the representative samples of injured arteries from the NIK^aly/aly^ and NIK^+/+^ control mice with 20x magnifications. (**B**) Quantification of the % of stenosis. (**C**) Quantification of the intima/media (I/M) ratio. Corresponding values for the sham NIK^aly/aly^ and NIK^+/+^ mice were 0 ± 0 for both stenosis and I/M ratio. ** *p* < 0.005; *** *p* < 0.0005, vs. NIK^+/+^ injured. Mean ± SEM of 4–6 mice per group.

To characterize the role of NIK during endothelial injury, injured femoral arteries from wild-type mice were studied as a control group (NIK^+/+^) two weeks after guidewire-induce endothelial denudation and compared with injured femoral arteries from NIK-deficient homozygous mice (NIK^aly/aly^) analyzed at the same time point. In hematoxylin-eosin (HE)-stained sections, the sham-operated arteries had a normal appearance. Following the endothelial denudation, intimal hyperplasia developed in at least one sample of every arterial segment. The injury was milder in the arteries from the NIK-deficient homozygous mice (NIK^aly/aly^) than in the arteries from the wild-type (NIK^+/+^) mice, as assessed by the intimal hyperplasia resulting in an increased intima/media (I/M) ratio (NIK^+/+^ 0.93 ± 0.53 vs. NIK^aly/aly^ 0.26 ± 0.02) and % stenosis (NIK^+/+^ 92.01 ± 0.74 vs. NIK^aly/aly^ 34.32 ± 5.37) (Figure 2).

**Figure 3:**
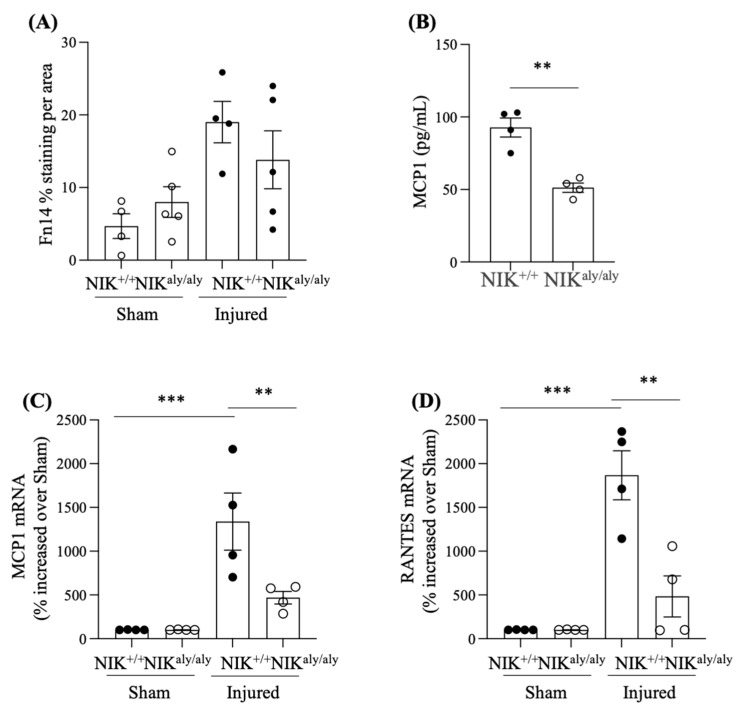
Decreased chemokine expression during vascular injury in NIK^aly/aly^ mice. The injured femoral arteries were studied two weeks following endothelial cell injury induced by a guidewire. (**A**) Fn14 mRNA increased in the injured arteries relative to the healthy arteries in the WT mice. There were no significant differences between the injured NIK^aly/aly^ and injured WT arteries. Data expressed as % increase over the contralateral control artery. (**B**) Circulating MCP1 levels (pg/mL) were lower in the NIK^aly/aly^ than in the WT mice. ** *p* < 0.005. MCP-1 (**C**) and RANTES (**D**) mRNA increased in the injured arteries compared to the healthy arteries in the WT mice and were lower in the injured arteries of the NIK^aly/aly^ than of the WT mice. ** *p* < 0.005; *** *p* < 0.0005, vs. NIK^+/+^ sham or NIK^+/+^ injured. Data expressed as % increase in the injured over the contralateral sham control artery. Mean ± SEM of 4–5 mice per group.

However, no differences in Fn14 immunostaining were observed between the injured NIK^aly/aly^ and injured WT NIK^+/+^ arteries (Figure 3A; NIK^+/+^ injured 19.03 ± 3.30 vs. NIK^aly/aly^ injured 16.22 ± 4.73% staining), i.e., the increased expression of the TWEAK receptor during injury appeared to be NIK-independent, which would be aligned with the current understanding of the system in which receptor activation is situated upstream of NIK activation [11,13].

### 2.3. NIK Deficiency Decreases Chemokine Expression

Next, we compared the inflammatory gene expression between the WT NIK^+/+^ and NIK^aly/aly^ mice.

However, circulating MCP-1 levels, evidence of systemic inflammation, were lower in the NIK^aly/aly^ than in the WT mice (Figure 3B; NIK^+/+^ 79.77 ± 5.18 vs. NIK^aly/aly^ 51.25 ± 3.19).

In this regard, the increased gene expression of the MCP-1 and RANTES chemokines observed in the injured WT arteries was significantly milder in the injured NIK^aly/aly^ than in the injured WT NIK^+/+^ arteries (Figure 3C,D; MCP1: NIK^+/+^ injured 1337.52 ± 325.04 vs. NIK^aly/aly^ injured 468.83 ± 72.18% increased; RANTES: NIK^+/+^ injured 1866.94 ± 280.68 vs. NIK^aly/aly^ injured 483.47 ± 234.64% increased.

Overall, these results are consistent with a NIK-independent upregulation of Fn14 expression following arterial injury and a blockade of NIK-mediated signaling from Fn14 to target genes such as those encoding chemokines when NIK was deficient.

### 2.4. NIK Deficiency Decreases Arterial Inflammation

**Figure 4:**
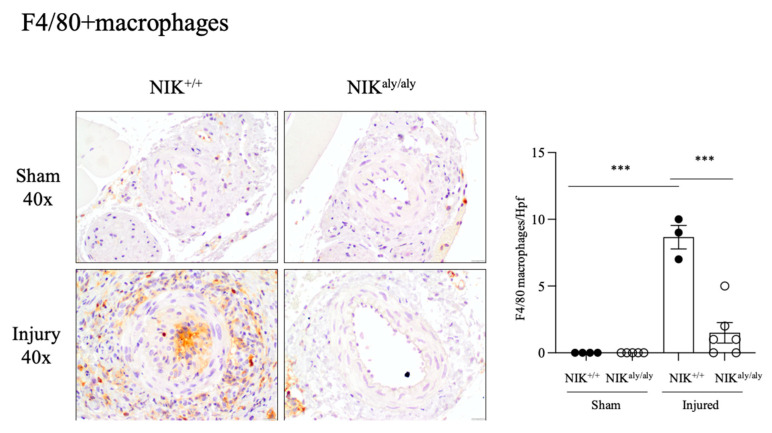
Infiltration by F4/80+ macrophages in mouse femoral arteries. The injured femoral arteries were studied two weeks following endothelial cell injury induced by a guidewire. The representative sections (40x magnification) and quantification of the sham or injured arteries. Leukocyte infiltration was mainly neointimal and periadventitial and more marked in the WT NIK^+/+^ than in the NIK^aly/aly^ mice. *** *p* < 0.0005, vs. NIK^+/+^ sham or NIK^+/+^ injured. Mean ± SEM of 4–5 mice per group.

In the injured arteries, an increased number of F4/80+ leukocytes was observed, located mainly in the intimal proliferation and adventitia, which was milder in the injured NIK-deficient NIK^aly/aly^ than in the injured WT NIK^+/+^ arteries (Figure 4; NIK^+/+^ injured 8.6 ± 0.88 vs. NIK^aly/aly^ injured 1.5 ± 0.76).

## 3. Discussion

We observed a key role for NIK in intimal hyperplasia and vessel stenosis induced by endothelial denudation-induced vascular injury. Following a literature review, we believe this finding is novel and not previously reported and may facilitate the development of novel therapeutic approaches. In this regard, small molecule NIK inhibitors are being developed and have been shown to be protective in liver injury and fibrosis, lymphoma, melanoma, and systemic lupus erythematosus in preclinical models [14,20,21,22,23,24,25,26,27].

Arterial injury following endothelial denudation was characterized by arterial stenosis, increased MCP1 and RANTES chemokines in the arterial wall, systemic inflammation, and arterial leukocyte infiltrates. Mice deficient in the proinflammatory kinase NIK were protected from the arterial stenosis caused by the endothelial injury. The mechanism of this protective effect could be a decrease in the inflammatory response, since NIK-deficient mice presented a lower expression of chemokine mRNA, as well as circulating MCP1 and milder macrophage infiltration. NIK is required for the non-canonical activation of the proinflammatory transcription factor NFκB in response to several cytokines of the TNF superfamily binding to their receptors, including TNFSFR12A (Fn14, Tweak receptor), lymphotoxin β receptor (LTβR), B-cell activating factor receptor (BAFF-R), the receptor activator of NF-κB (RANK), CD40, and CD27 and to other stimuli such as LPS [28,29]. In this regard, classical NFκB activation is usually a rapid and transient response to a wide range of stimuli whose main effector is RelA/p50 while the non-canonical NFκB pathway activation results in delayed and prolonged responses to a smaller range of stimuli resulting in the DNA binding of RelB/p52 complexes [11]. While we did not perform intervention experiments that identified the specific ligands that activate NIK in the context of intimal hyperplasia following endothelial denudation-induced vascular injury, we did observe an increased expression of the Fn14 receptor. The upregulation of Fn14 gene expression is known to be the main pathway for the activation of the TWEAK/Fn14 axis [19,30]. Furthermore, Fn14 activation causes cardiovascular injury and is considered a major therapeutic target for post-angioplasty restenosis and atherosclerosis [31,32]. Overall, the results were consistent with a NIK-independent upregulation of Fn14 expression following endothelial injury and the blockade of NIK-mediated signaling from Fn14 to target genes such as those encoding chemokines in NIK-deficient mice. This observation is consistent with the known role of NIK in mediating the Fn14-induced upregulation of the proinflammatory transcription factor NFκB and downstream chemokines [11,13].

Contrary to chronic conditions in which NIK targeting may require prolonged treatment, thus increasing the risk of adverse effects, we have now shown that NIK deficiency prevents the adverse consequences of endothelial injury over two weeks. Thus, the data are consistent with a protective role of short-term NIK inhibition around vascular interventions to prevent endothelial injury and the subsequent intimal hyperplasia and stenosis. Eventually, NIK inhibitors may be embedded in stents to prevent vascular stenosis, as performed for other drugs [33]. Clinical trials should address these possibilities. By contrast, long-term NIK inhibition is associated with immune deficiency: Patients with autosomal recessive NIK deficiency or deficiency in the other members of the pathway such as RELB or NFκB2 may develop neutralizing autoantibodies against type I interferons and are at higher risk of life-threatening COVID-19 pneumonia [34]. This human phenotype is consistent with the immune deficiency found in NIK^aly/aly^ mice [35].

While targeting NIK had not been previously explored to address the function of NIK in endothelial denudation-induced arterial stenosis, our data are consistent with prior reports in the literature suggesting an involvement of NIK in vascular injury. Most of these reports addressed the role of NIK by functional targeting in endothelial injury and endothelial stress-triggered inflammatory responses in cultured endothelial cells [36,37,38,39,40]. One publication reported on the NIK facilitation of high glucose-induced proinflammatory responses in cultured endothelial cells [40]. In vivo, one report demonstrated that the endothelial-specific deficiency of NIK IRF-1 attenuated atherosclerosis in high-fat diet-fed Apoe-null mice [39], and another indicated the need for NIK in lymphatic endothelial cells to activate the non-canonical NFκB pathway to regulate B-cell homing to lymph nodes [41]. These reports also identified NIK as required for the complement membrane attack complex-induced inflammatory responses in endothelial cells through the activation of non-canonical NFκB signaling [36,37]. NIK also contributed to the activation of NFκB by flow in cultured human endothelial cells [42]. In clinical human samples, NIK+ endothelial cells were observed in atherosclerotic lesions, together with the transcriptomic evidence of non-canonical NFκB activation [43].

Although NIK inhibition has not yet reached clinical development, there is very active research on effective and safe NIK inhibitors [20,21,44,45,46,47,48]. In this regard, recent research has emphasized the involvement of NIK in metabolic disease [49,50,51], sarcopenia [52], liver disease [22,53,54], malignancy [55,56,57,58,59,60,61,62,63,64,65], sepsis [46], neurological disease [45], and inflammation of multiple organs [66], including immune-mediated vasculitis and complement-mediated endothelial cell injury [37,48,67].

Some limitations should be acknowledged. This is a preclinical study, and no intervention was assessed in human subjects.

In conclusion, NIK deficiency decreased intimal hyperplasia and vessel stenosis induced by endothelial denudation-induced vascular injury in vivo and reduced local and systemic inflammatory responses. These results are aligned with prior evidence for the role of NIK in triggering proinflammatory responses in cultured endothelial cells and the role of NIK in preclinical atherosclerosis. Given the advances in the development of NIK inhibitors, the present data provide a rationale for the design of clinical trials targeting NIK in the peri-intervention period to improve outcomes in vascular grafts while minimizing the immunosuppressive effect of NIK targeting. Stents eluting NIK inhibitors could be tested for that purpose.

## 4. Materials and Methods

### 4.1. Experimental Model

Animal studies were carried out in accordance with the Guidelines of the National Institutes of Health for the Care and Use of Laboratory Animals, respecting the current national regulations. To explore the role of NIK in intimal hyperplasia, 10-week-old male NIK^aly/aly^ mice and wild-type NIK^+/+^ littermate control mice from the Center for Molecular Biology Severo Ochoa (CBMSO, Madrid, Spain) animal facilities [12] were studied (n = 4–6 per group). The NIK^aly/aly^ mice have deficient NIK activity due to a point mutation (NIK^aly^ mutation) that causes an amino acid substitution in the carboxy terminal interaction domain of NIK that inactivates the enzyme [12,68]. The mice were anesthetized by ketamine/xylazine and endoluminal injury to the common femoral artery was performed by three passages of a 0.25 mm diameter angioplasty guidewire (103-0601-300: ev3 X-celerator^TM^-10 Exchange Hydrophilic Guidewire: 0.010″ × 300 cm, Medtronic) as previously described [69]. In control arteries, a similar procedure was simulated but the guidewire was not inserted [70]. The surgical time for bilateral femoral artery intervention was 10 to 20 min per mouse. The mice were sacrificed 14 days after the wire injury by administering 5 mg/100 g sodium pentobarbital and plasma samples and femoral artery samples were collected. The tissue was fixed and used for immunohistochemistry or snap-frozen in liquid nitrogen for RNA studies.

### 4.2. Histomorphometry

The femoral artery was excised from the inguinal ligament to the branching of the profundal femoris artery and stored in paraformaldehyde for 24 h and later in ethanol (Merck, Darmstadt, Germany) until paraffin embedded. Sections 2 µm thick were mounted on slides pretreated with 2% APES (Sigma A3648, Burlington, Massachusetts, MA, USA) in acetone. To ensure an equitable and random distribution of representative samples, 10 segments of each artery were studied and the numerical results represent the average injury over the entire artery and are not limited to a single area (Figure S1). From each segment, 6 sections were obtained, one for hematoxylin-eosin (Merck, Darmstadt, Germany) staining for the histological and histomorphometric studies, and the rest for the immunohistochemical studies. The samples with complete thrombosis were discarded.

In the hematoxylin-stained samples, the areas of the vessel lumen, intimal hyperplasia, and arterial media were quantitated using the Image Pro Plus quantitative image analysis system (Media Cybernetics, Rockville, MD 20852, USA). Intimal hyperplasia was defined as any proliferative lesion within the circumference of the internal elastic lamina. The percentage of stenosis (% stenosis = 100 × [Intimal area/(Lumen area + Intimal area)]) and the intima/media ratio (I/M = Intimal area/Media area) were calculated using the formula described in previous publications [69,71,72]: The arteries with the sham surgery, as they did not present proliferative lesions, were assigned an I/M of zero.

### 4.3. Immunohistochemistry

Immunohistochemistry was performed as previously described on paraffin-embedded 2 µm thick tissue sections [73]. The primary antibodies were rabbit anti-NIK (1:100, Cell Signaling, Danvers, MA, USA), rabbit anti-Fn14 (Cell Signaling, Danvers, MA, USA), and polyclonal rat anti-F4/80 antibody (Serotec, Oxford, UK, 1:50). The sections were counterstained with Carazzi’s hematoxylin. The negative controls included incubation with a non-specific immunoglobulin of the same isotype as the primary antibody.

### 4.4. RNA Expression Studies: Reverse Transcription and Real-Time PCR

Total RNA was extracted following the instructions of the commercial “RNeasy Micro Kit” (Qiagen, Hilden, Germany), which is designed to purify up to 45 µg RNA from small tissue samples. Reverse transcription was performed with 1 µg of RNA using the High Capacity cDNA Archive Kit (Applied Biosystems, Foster City, CA, USA) as previously described [74]. Real-time PCR was performed by the ABI Prism 7500 Sequential Detection System (v1.5.1) predeveloped primers (Applied Biosystems, Foster City, CA, USA). In parallel to the gene of interest, GAPDH mRNA was amplified as an internal control. All the measurements were performed in duplicate.

### 4.5. ELISA

Plasma MCP-1 was assessed in duplicate samples (100 µL plasma) using a commercial ELISA kit (BD Pharmingen, San Diego, CA). The assay was calibrated using a standard curve with known concentrations of soluble MCP-1. Assay range: 3.9–250 pg/mL.

### 4.6. Statistical Analysis

Statistical analysis was performed using the GraphPad Prism Software 8 (CA, USA). The results are expressed as mean ± SEM. Significance at the *p* < 0.05 level was assessed by the Student’s *t*-test or Mann–Whitney test for two groups of data. For three or more groups, an ANOVA followed by Bonferroni post hoc correction or the Kruskal–Wallis test followed by the Mann–Whitney test were performed. *p*-values were coded as follows: * *p* < 0.05; ** *p* < 0.005; *** *p* < 0.0005.

## Data Availability

The data used and/or analyzed during the current study are available from the corresponding author upon reasonable request.

## Supplementary Materials

The following supporting information can be downloaded at: https://www.mdpi.com/article/10.3390/ijms252111473/s1.

## References

[1] Foreman K.J., Marquez N., Dolgert A., Fukutaki K., Fullman N., McGaughey M., Pletcher M.A., Smith A.E., Tang K., Yuan C.W. (2018). Forecasting Life Expectancy, Years of Life Lost, and All-Cause and Cause-Specific Mortality for 250 Causes of Death: Reference and Alternative Scenarios for 2016-40 for 195 Countries and Territories. Lancet.

[2] Epstein F.H., Gibbons G.H., Dzau V.J. (1994). The Emerging Concept of Vascular Remodeling. N. Engl. J. Med..

[3] Schwartz S.M., DeBlois D., O’Brien E.R.M. (1995). The Intima. Soil for Atherosclerosis and Restenosis. Circ. Res..

[4] Pashova A., Work L.M., Nicklin S.A. (2020). The Role of Extracellular Vesicles in Neointima Formation Post Vascular Injury. Cell. Signal..

[5] Moriarty J.P., Murad M.H., Shah N.D., Prasad C., Montori V.M., Erwin P.J., Forbes T.L., Meissner M.H., Stoner M.C. (2011). A Systematic Review of Lower Extremity Arterial Revascularization Economic Analyses. J. Vasc. Surg..

[6] Dosluoglu H.H., Lall P., Harris L.M., Dryjski M.L. (2012). Long-Term Limb Salvage and Survival after Endovascular and Open Revascularization for Critical Limb Ischemia after Adoption of Endovascular-First Approach by Vascular Surgeons. J. Vasc. Surg..

[7] Gimbrone M.A., García-Cardeña G. (2016). Endothelial Cell Dysfunction and the Pathobiology of Atherosclerosis. Circ. Res..

[8] Reiss A.B., Jacob B., Ahmed S., Carsons S.E., DeLeon J. (2021). Understanding Accelerated Atherosclerosis in Systemic Lupus Erythematosus: Toward Better Treatment and Prevention. Inflammation.

[9] Mussbacher M., Salzmann M., Brostjan C., Hoesel B., Schoergenhofer C., Datler H., Hohensinner P., Basílio J., Petzelbauer P., Assinger A. (2019). Cell Type-Specific Roles of NF-ΚB Linking Inflammation and Thrombosis. Front. Immunol..

[10] Sanz A.B., Sanchez-Niño M.D., Ramos A.M., Moreno J.A., Santamaria B., Ruiz-Ortega M., Egido J., Ortiz A. (2010). NF-KappaB in Renal Inflammation. J. Am. Soc. Nephrol..

[11] Valiño-Rivas L., Vaquero J.J., Sucunza D., Gutierrez S., Sanz A.B., Fresno M., Ortiz A., Sanchez-Niño M.D. (2019). NIK as a Druggable Mediator of Tissue Injury. Trends Mol. Med..

[12] Ortiz A., Husi H., Gonzalez-Lafuente L., Valiño-Rivas L., Fresno M., Sanz A.B., Mullen W., Albalat A., Mezzano S., Vlahou T. (2017). Mitogen-Activated Protein Kinase 14 Promotes AKI. J. Am. Soc. Nephrol..

[13] Cuarental L., Sucunza-Sáenz D., Valiño-Rivas L., Fernandez-Fernandez B., Sanz A.B., Ortiz A., Vaquero J.J., Sanchez-Niño M.D. (2019). MAP3K Kinases and Kidney Injury. Nefrologia.

[14] Brightbill H.D., Suto E., Blaquiere N., Ramamoorthi N., Sujatha-Bhaskar S., Gogol E.B., Castanedo G.M., Jackson B.T., Kwon Y.C., Haller S. (2018). NF-ΚB Inducing Kinase Is a Therapeutic Target for Systemic Lupus Erythematosus. Nat. Commun..

[15] Taylor T., Anastasiou C., Ja C., Rush S., Trupin L., Dall’Era M., Katz P., Barbour K.E., Greenlund K.J., Yazdany J. (2023). Causes of Death Among Individuals With Systemic Lupus Erythematosus by Race and Ethnicity: A Population-Based Study. Arthritis Care Res..

[16] Poveda J., Tabara L.C., Fernandez-Fernandez B., Martin-Cleary C., Sanz A.B., Selgas R., Ortiz A., Sanchez-Niño M.D. (2013). TWEAK/Fn14 and Non-Canonical NF-KappaB Signaling in Kidney Disease. Front. Immunol..

[17] Sastre C., Fernández-Laso V., Madrigal-Matute J., Muñoz-García B., Moreno J.A., Pastor-Vargas C., Llamas-Granda P., Burkly L.C., Egido J., Martín-Ventura J.L. (2014). Genetic Deletion or TWEAK Blocking Antibody Administration Reduce Atherosclerosis and Enhance Plaque Stability in Mice. J. Cell. Mol. Med..

[18] Muñoz-García B., Moreno J.A., López-Franco O., Sanz A.B., Martín-Ventura J.L., Blanco J., Jakubowski A., Burkly L.C., Ortiz A., Egido J. (2009). Tumor Necrosis Factor-like Weak Inducer of Apoptosis (TWEAK) Enhances Vascular and Renal Damage Induced by Hyperlipidemic Diet in ApoE-Knockout Mice. Arterioscler. Thromb. Vasc. Biol..

[19] Sanz A.B., Izquierdo M.C., Sanchez-Niño M.D., Ucero A.C., Egido J., Ruiz-Ortega M., Ramos A.M., Putterman C., Ortiz A. (2014). TWEAK and the Progression of Renal Disease: Clinical Translation. Nephrol. Dial. Transpl..

[20] Tang Y. (2023). Analysis of the Binding Pattern of NIK Inhibitors by Computational Simulation. J. Biomol. Struct. Dyn..

[21] Crawford J.J., Feng J., Brightbill H.D., Johnson A.R., Wright M., Kolesnikov A., Lee W., Castanedo G.M., Do S., Blaquiere N. (2023). Filling a Nick in NIK: Extending the Half-Life of a NIK Inhibitor through Structure-Based Drug Design. Bioorg. Med. Chem. Lett..

[22] Zhang Z., Zhong X., Shen H., Sheng L., Liangpunsakul S., Lok A.S., Omary M.B., Wang S., Rui L. (2022). Biliary NIK Promotes Ductular Reaction and Liver Injury and Fibrosis in Mice. Nat. Commun..

[23] Zhu Y., Ma Y., Zu W., Song J., Wang H., Zhong Y., Li H., Zhang Y., Gao Q., Kong B. (2020). Identification of N-Phenyl-7 H-Pyrrolo[2,3- d]Pyrimidin-4-Amine Derivatives as Novel, Potent, and Selective NF-ΚB Inducing Kinase (NIK) Inhibitors for the Treatment of Psoriasis. J. Med. Chem..

[24] Mondragón L., Mhaidly R., De Donatis G.M., Tosolini M., Dao P., Martin A.R., Pons C., Chiche J., Jacquin M., Imbert V. (2019). GAPDH Overexpression in the T Cell Lineage Promotes Angioimmunoblastic T Cell Lymphoma through an NF-ΚB-Dependent Mechanism. Cancer Cell.

[25] Cheng G., Mei X.B., Yan Y.Y., Chen J., Zhang B., Li J., Dong X.W., Lin N.M., Zhou Y.B. (2019). Identification of New NIK Inhibitors by Discriminatory Analysis-Based Molecular Docking and Biological Evaluation. Arch. Pharm..

[26] Pippione A.C., Sainas S., Federico A., Lupino E., Piccinini M., Kubbutat M., Contreras J.M., Morice C., Barge A., Ducime A. (2018). N-Acetyl-3-Aminopyrazoles Block the Non-Canonical NF-KB Cascade by Selectively Inhibiting NIK. Medchemcomm.

[27] Takeda T., Tsubaki M., Sakamoto K., Ichimura E., Enomoto A., Suzuki Y., Itoh T., Imano M., Tanabe G., Muraoka O. (2016). Mangiferin, a Novel Nuclear Factor Kappa B-Inducing Kinase Inhibitor, Suppresses Metastasis and Tumor Growth in a Mouse Metastatic Melanoma Model. Toxicol. Appl. Pharmacol..

[28] Sun S.C. (2012). The Noncanonical NF-ΚB Pathway. Immunol. Rev..

[29] Pflug K.M., Sitcheran R. (2020). Targeting NF-ΚB-Inducing Kinase (NIK) in Immunity, Inflammation, and Cancer. Int. J. Mol. Sci..

[30] Ratajczak W., Atkinson S.D., Kelly C. (2022). The TWEAK/Fn14/CD163 Axis-Implications for Metabolic Disease. Rev. Endocr. Metab. Disord..

[31] Méndez-Barbero N., Gutierrez-Muñoz C., Madrigal-Matute J., Mínguez P., Egido J., Michel J.B., Martín-Ventura J.L., Esteban V., Blanco-Colio L.M. (2019). A Major Role of TWEAK/Fn14 Axis as a Therapeutic Target for Post-Angioplasty Restenosis. EBioMedicine.

[32] Fernández-Laso V., Sastre C., Méndez-Barbero N., Egido J., Martín-Ventura J.L., Gómez-Guerrero C., Blanco-Colio L.M. (2017). TWEAK Blockade Decreases Atherosclerotic Lesion Size and Progression through Suppression of STAT1 Signaling in Diabetic Mice. Sci. Rep..

[33] Zhao K., Zeng Z., He Y., Zhao R., Niu J., Sun H., Li S., Dong J., Jing Z., Zhou J. (2024). Recent Advances in Targeted Therapy for Inflammatory Vascular Diseases. J. Control Release.

[34] Le Voyer T., Parent A.V., Liu X., Cederholm A., Gervais A., Rosain J., Nguyen T., Perez Lorenzo M., Rackaityte E., Rinchai D. (2023). Autoantibodies against Type I IFNs in Humans with Alternative NF-ΚB Pathway Deficiency. Nature.

[35] Shinkura R., Kitada K., Matsuda F., Tashiro K., Ikuta K., Suzuki M., Kogishi K., Serikawa T., Honjo T. (1999). Alymphoplasia Is Caused by a Point Mutation in the Mouse Gene Encoding Nf-Kappa b-Inducing Kinase. Nat. Genet..

[36] Jane-wit D., Surovtseva Y.V., Qin L., Li G., Liu R., Clark P., Manes T.D., Wang C., Kashgarian M., Kirkiles-Smith N.C. (2015). Complement Membrane Attack Complexes Activate Noncanonical NF-ΚB by Forming an Akt+ NIK+ Signalosome on Rab5+ Endosomes. Proc. Natl. Acad. Sci. USA.

[37] Xie C.B., Qin L., Li G., Fang C., Kirkiles-Smith N.C., Tellides G., Pober J.S., Jane-Wit D. (2019). Complement Membrane Attack Complexes Assemble NLRP3 Inflammasomes Triggering IL-1 Activation of IFN-γ-Primed Human Endothelium. Circ. Res..

[38] Kucharzewska P., Maracle C.X., Jeucken K.C.M., Van Hamburg J.P., Israelsson E., Furber M., Tas S.W., Olsson H.K. (2019). NIK-IKK Complex Interaction Controls NF-ΚB-Dependent Inflammatory Activation of Endothelium in Response to LTβR Ligation. J. Cell Sci..

[39] Fan X., Li Q., Wang Y., Zhang D.M., Zhou J., Chen Q., Sheng L., Passerini A.G., Sun C.X. (2023). Non-Canonical NF-ΚB Contributes to Endothelial Pyroptosis and Atherogenesis Dependent on IRF-1. Transl. Res..

[40] Li B., Li H., Dai L., Liu C., Wang L., Li Q., Gu C. (2022). NIK-SIX1 Signalling Axis Regulates High Glucose-Induced Endothelial Cell Dysfunction and Inflammation. Autoimmunity.

[41] Yang J., Zhang S., Zhang L., Xie X., Wang H., Jie Z., Gu M., Yang J.Y., Cheng X., Sun S.C. (2019). Lymphatic Endothelial Cells Regulate B-Cell Homing to Lymph Nodes via a NIK-Dependent Mechanism. Cell. Mol. Immunol..

[42] Hay D.C., Beers C., Cameron V., Thomson L., Flitney F.W., Hay R.T. (2003). Activation of NF-ΚB Nuclear Transcription Factor by Flow in Human Endothelial Cells. Biochim. Biophys. Acta Mol. Cell Res..

[43] Maracle C.X., Agca R., Helder B., Meeuwsen J.A.L., Niessen H.W.M., Biessen E.A.L., de Winther M.P.J., de Jager S.C.A., Nurmohamed M.T., Tas S.W. (2018). Noncanonical NF-ΚB Signaling in Microvessels of Atherosclerotic Lesions Is Associated with Inflammation, Atheromatous Plaque Morphology and Myocardial Infarction. Atherosclerosis.

[44] Anderson J.J., Grillo M.J., Harki D.A. (2023). Development of Allosteric NIK Ligands from Fragment-Based NMR Screening. ACS Med. Chem. Lett..

[45] Cao M., Yi L., Xu Y., Tian Y., Li Z., Bi Y., Guo M., Li Y., Liu Y., Xu X. (2024). Inhibiting NF-ΚB Inducing Kinase Improved the Motor Performance of ALS Animal Model. Brain Res..

[46] Zhang N., Shen S., Yang M., He S., Liu C., Li H., Lu T., Liu H., Hu Q., Tang W. (2024). Design, Synthesis, and Biological Evaluation of a Novel NIK Inhibitor with Anti-Inflammatory and Hepatoprotective Effects for Sepsis Treatment. J. Med. Chem..

[47] Zhang K., Tang Y., Yu H., Yang J., Tao L., Xiang P. (2024). Discovery of Lupus Nephritis Targeted Inhibitors Based on De Novo Molecular Design: Comprehensive Application of Vinardo Scoring, ADMET Analysis, and Molecular Dynamics Simulation. J. Biomol. Struct. Dyn..

[48] Merino-Vico A., van Hamburg J.P., Tuijnenburg P., Frazzei G., Al-Soudi A., Bonasia C.G., Helder B., Rutgers A., Abdulahad W.H., Stegeman C.A. (2024). Targeting NF-ΚB Signaling in B Cells as a Potential New Treatment Modality for ANCA-Associated Vasculitis. J. Autoimmun..

[49] Chen X., Liu Z., Liu W., Wang S., Jiang R., Hu K., Sheng L., Xu G., Kou X., Song Y. (2023). NF-ΚB-Inducing Kinase Provokes Insulin Resistance in Skeletal Muscle of Obese Mice. Inflammation.

[50] Pflug K.M., Lee D.W., Keeney J.N., Sitcheran R. (2023). NF-ΚB-Inducing Kinase Maintains Mitochondrial Efficiency and Systemic Metabolic Homeostasis. Biochim. Biophys. Acta. Mol. Basis Dis..

[51] Mori K., Mizokami A., Sano T., Mukai S., Hiura F., Ayukawa Y., Koyano K., Kanematsu T., Jimi E. (2022). RANKL Elevation Activates the NIK/NF-ΚB Pathway, Inducing Obesity in Ovariectomized Mice. J. Endocrinol..

[52] Bilgic S.N., Domaniku A., Toledo B., Agca S., Weber B.Z.C., Arabaci D.H., Ozornek Z., Lause P., Thissen J.P., Loumaye A. (2023). EDA2R-NIK Signalling Promotes Muscle Atrophy Linked to Cancer Cachexia. Nature.

[53] Xu C., Zhou H., Jin Y., Sahay K., Robicsek A., Liu Y., Dong K., Zhou J., Barrett A., Su H. (2022). Hepatic Neddylation Deficiency Triggers Fatal Liver Injury via Inducing NF-ΚB-Inducing Kinase in Mice. Nat. Commun..

[54] Vesting A.J., Jais A., Klemm P., Steuernagel L., Wienand P., Fog-Tonnesen M., Hvid H., Schumacher A.L., Kukat C., Nolte H. (2022). NIK/MAP3K14 in Hepatocytes Orchestrates NASH to Hepatocellular Carcinoma Progression via JAK2/STAT5 Inhibition. Mol. Metab..

[55] Xu K., Kessler A., Nichetti F., Hoffmeister-Wittmann P., Scherr A.L., Nader L., Kelmendi E., Schmitt N., Schwab M., García-Beccaria M. (2024). Lymphotoxin Beta-Activated LTBR/NIK/RELB Axis Drives Proliferation in Cholangiocarcinoma. Liver Int..

[56] Jung D.E., Seo M.K., Jo J.H., Kim K., Kim C., Kang H., Park S.B., Lee H.S., Kim S., Song S.Y. (2024). PUM1-TRAF3 Fusion Protein Activates Non-Canonical NF-ΚB Signaling via Rescued NIK in Biliary Tract Cancer. NPJ Precis. Oncol..

[57] Decombis S., Papin A., Bellanger C., Sortais C., Dousset C., Le Bris Y., Riveron T., Blandin S., Hulin P., Tessoulin B. (2022). The IL32/BAFF Axis Supports Prosurvival Dialogs in the Lymphoma Ecosystem and Is Disrupted by NIK Inhibition. Haematologica.

[58] Agca S., Kir S. (2024). EDA2R-NIK Signaling in Cancer Cachexia. Curr. Opin. Support. Palliat. Care.

[59] Morrison H.A., Eden K., Trusiano B., Rothschild D.E., Qin Y., Wade P.A., Rowe A.J., Mounzer C., Stephens M.C., Hanson K.M. (2024). NF-ΚB Inducing Kinase Attenuates Colorectal Cancer by Regulating Noncanonical NF-ΚB Mediated Colonic Epithelial Cell Regeneration. Cell. Mol. Gastroenterol. Hepatol..

[60] Li M.Y., Chong L.C., Duns G., Lytle A., Woolcock B., Jiang A., Telenius A., Ben-Neriah S., Nawaz W., Slack G.W. (2024). TRAF3 Loss-of-Function Reveals the Noncanonical NF-ΚB Pathway as a Therapeutic Target in Diffuse Large B Cell Lymphoma. Proc. Natl. Acad. Sci. USA.

[61] Cormier F., Housni S., Dumont F., Villard M., Cochand-Priollet B., Mercier-Nomé F., Perlemoine K., Bertherat J., Groussin L. (2023). NF-ΚB Signaling Activation and Roles in Thyroid Cancers: Implication of MAP3K14/NIK. Oncogenesis.

[62] Pflug K.M., Lee D.W., Tripathi A., Bankaitis V.A., Burgess K., Sitcheran R. (2023). Cyanine Dye Conjugation Enhances Crizotinib Localization to Intracranial Tumors, Attenuating NF-ΚB-Inducing Kinase Activity and Glioma Progression. Mol. Pharm..

[63] Pflug K.M., Lee D.W., McFadden K., Herrera L., Sitcheran R. (2023). Transcriptional Induction of NF-ΚB-Inducing Kinase by E2F4/5 Facilitates Collective Invasion of GBM Cells. Sci. Rep..

[64] Daren L., Dan Y., Jinhong W., Chao L. (2024). NIK-Mediated Reactivation of SIX2 Enhanced the CSC-like Traits of Hepatocellular Carcinoma Cells through Suppressing Ubiquitin-Proteasome System. Environ. Toxicol..

[65] Hayashi Y., Nakayama J., Yamamoto M., Maekawa M., Watanabe S., Higashiyama S., Inoue J.I., Yamamoto Y., Semba K. (2023). Aberrant Accumulation of NIK Promotes Tumor Growth by Dysregulating Translation and Post-Translational Modifications in Breast Cancer. Cancer Cell Int..

[66] Xia X., Zhu L., Xu M., Lei Z., Yu H., Li G., Wang X., Jia H., Yin Z., Huang F. (2024). ANKRD22 Promotes Resolution of Psoriasiform Skin Inflammation by Antagonizing NIK-Mediated IL-23 Production. Mol. Ther..

[67] Xie C.B., Jiang B., Qin L., Tellides G., Kirkiles-Smith N.C., Jane-Wit D., Pober J.S. (2020). Complement-Activated Interferon-γ-Primed Human Endothelium Transpresents Interleukin-15 to CD8+ T Cells. J. Clin. Investig..

[68] Sánchez-Valdepeñas C., Martín A.G., Ramakrishnan P., Wallach D., Fresno M. (2006). NF-KappaB-Inducing Kinase Is Involved in the Activation of the CD28 Responsive Element through Phosphorylation of c-Rel and Regulation of Its Transactivating Activity. J. Immunol..

[69] Roque M., Fallon J.T., Badimon J.J., Zhang W.X., Taubman M.B., Reis E.D. (2000). Mouse Model of Femoral Artery Denudation Injury Associated with the Rapid Accumulation of Adhesion Molecules on the Luminal Surface and Recruitment of Neutrophils. Arterioscler. Thromb. Vasc. Biol..

[70] Baeza C., Pintor-Chocano A., Carrasco S., Sanz A., Ortiz A., Sanchez-Niño M.D. (2024). Paricalcitol Has a Potent Anti-Inflammatory Effect in Rat Endothelial Denudation-Induced Intimal Hyperplasia. Int. J. Mol. Sci..

[71] Gallo R., Padurean A., Toschi V., Bichler J., Fallon J.T., Chesebro J.H., Fuster V., Badimon J.J. (1998). Prolonged Thrombin Inhibition Reduces Restenosis after Balloon Angioplasty in Porcine Coronary Arteries. Circulation.

[72] Silvestre-Roig C., Fernández P., Esteban V., Pello M., Indolfi C., Rodríguez C., Rodríguez-Calvo R., López-Maderuelo M.D., Bauriedel G., Hutter R. (2013). Inactivation of Nuclear Factor-Y Inhibits Vascular Smooth Muscle Cell Proliferation and Neointima Formation. Arterioscler. Thromb. Vasc. Biol..

[73] Cuarental L., Ribagorda M., Ceballos M.I., Pintor-Chocano A., Carriazo S.M., Dopazo A., Vazquez E., Suarez-Alvarez B., Cannata-Ortiz P., Sanz A.B. (2023). The Transcription Factor Fosl1 Preserves Klotho Expression and Protects from Acute Kidney Injury. Kidney Int..

[74] Valiño-Rivas L., Cuarental L., Ceballos M.I., Pintor-Chocano A., Perez-Gomez M.V., Sanz A.B., Ortiz A., Sanchez-Niño M.D. (2022). Growth Differentiation Factor-15 Preserves Klotho Expression in Acute Kidney Injury and Kidney Fibrosis. Kidney Int..

