# Supplementary material for: NIK Is a Mediator of Inflammation and Intimal Hyperplasia in Endothelial Denudation-Induced Vascular Injury
Source: Int J Mol Sci. 2024 Oct 25;25(21):11473. doi: 10.3390/ijms252111473 (PMC11546836; doi:10.3390/ijms252111473)
Supplement: Supplementary file 1 [file ijms-25-11473-s001.zip › ijms-3218151-supplementary.pdf]

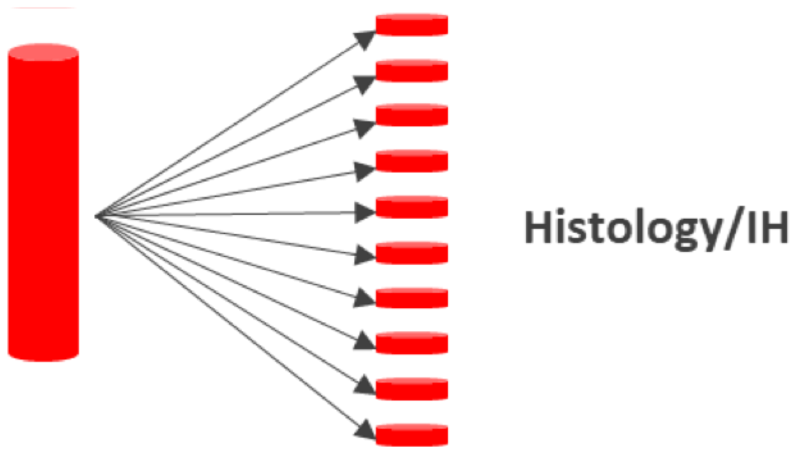

Figure S1. Experimental design. The entire femoral artery was divided into 10 consecutive segments that were divided into sections stained with different techniques and results averaged to yield the result of the sample.
